# Understanding form and function of the stem in early flattened echinoderms (pleurocystitids) using a microstructural approach

**DOI:** 10.7717/peerj.1820

**Published:** 2016-04-25

**Authors:** Przemysław Gorzelak, Samuel Zamora

**Affiliations:** 1 Department of Biogeology, Institute of Paleobiology, Polish Academy of Sciences, Warsaw, Poland; 2 Instituto Geológico y Minero de España, Zaragoza, Spain; 3 National Museum of Natural History, Smithsonian Institution, Washington, DC, USA

**Keywords:** Blastozoa, Stereom, Canada, Paleozoic, Palaeoanatomy, Rhombifera, Ordovician

## Abstract

Pleurocystitid rhombiferans are among the most unusual echinoderms whose mode of life has been long debated. These echinoderms are usually interpreted as vagile epibenthic echinoderms, moving over the sea bottom by means of a flexible stem. Although their life habits and posture are reasonably well understood, the mechanisms that control the movement of stem are highly controversial. Specifically, it is unknown whether the stem flexibility was under the control of muscles or ligamentary mutable collagenous tissues (MCTs). Here, we reconstruct palaeoanatomy of the two Ordovician pleurocystitid rhombiferans (*Pleurocystites* and *Amecystis*) based on stereom microstructure. We show that the articular facets of columnals in pleurocystitid rhombiferans are composed of fine labyrinthic stereom. Comparison with modern echinoderms suggests that this type of stereom was associated with muscles implying that their stem was a muscular locomotory organ supporting an active mode of life.

## Introduction

Early echinoderms exhibited disparate body plans including 30 different clades that first appeared in the Cambrian and Ordovician (Sumrall & Wray, 2007). Understanding the mode of life of some of these groups is controversial, especially those exhibiting aberrant body plans and lacking modern analogues (Clausen & Smith, 2005). Pleurocystitids (Pleurocystidae) are one of the most enigmatic echinoderms and are often illustrated in palaeontological textbooks as examples of flattened echinoderms. This echinoderm group belongs to the glyptocystitid rhombiferans (Gyptocystitida, Rhombifera, Blastozoa) and are known from the Early Ordovician to Middle Devonian (Nardin & Bohaty, 2013). The peculiar flattened body shape with two large feeding appendages in pleurocystitids is, however, considered as a derived condition within glyptocystitids (Sumrall & Sprinkle, 1995). Most glyptocystitids possess a globular theca that is composed of four plate circlets including: four basals (one of them, B4, is larger and located in the BC interradius), five infralaterals, five laterals, and four to ten radials. The summit is plated with seven interradial orals within the CD interradius divided into three orals (O1, O6 and O7) associated with the hydropore and gonopore (Sumrall & Sprinkle, 1999).

**Figure 1:**
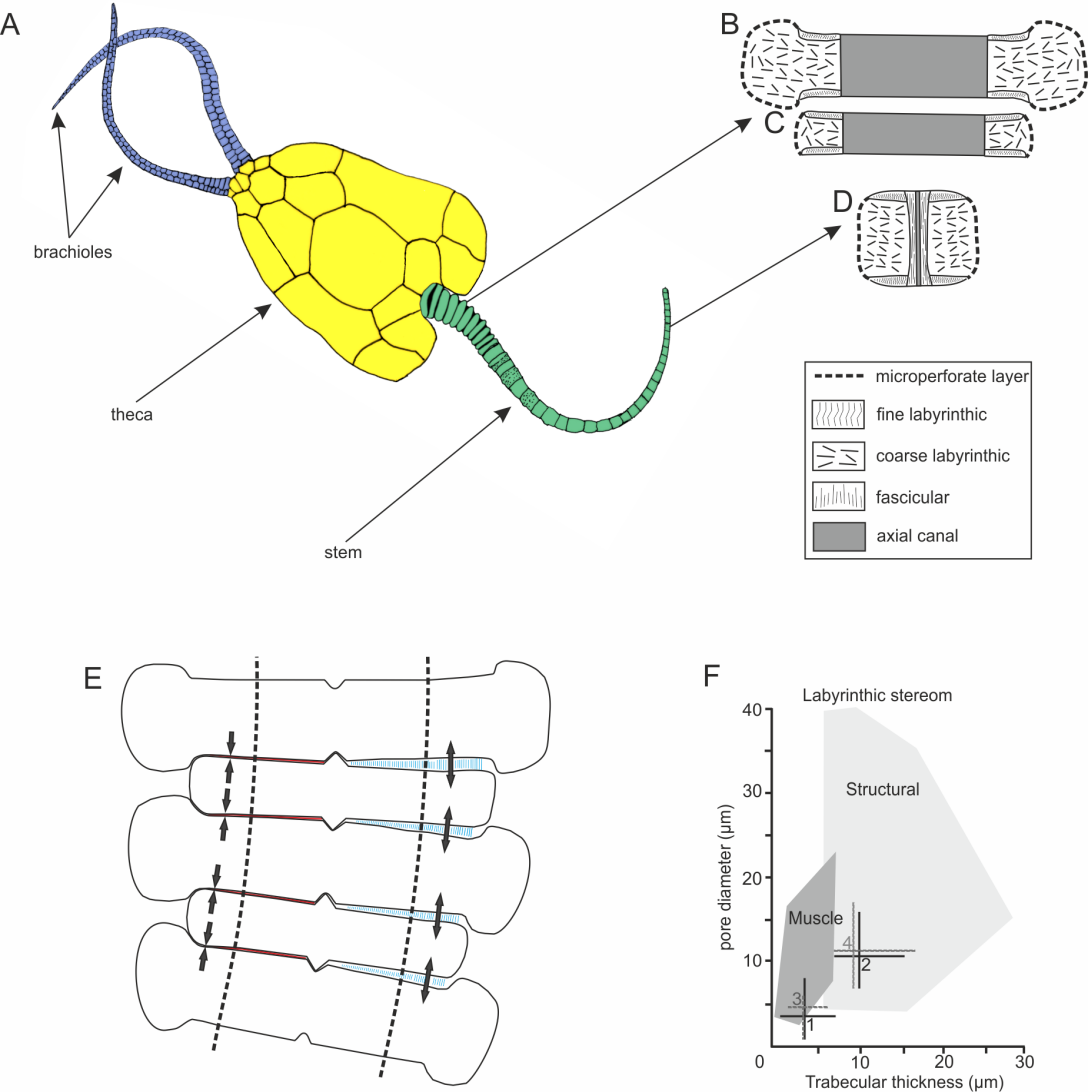
Morphology and microstructure of pleurocystitids. (A) pleurocystitid reconstruction (modified after Parsley, 1970); (B–D) stereom organization in outer proximal (B), inner proximal (C) and distal columnal (D); (E) functional model for pleurocystitid stem flexibility, muscles in relaxed (blue) and contracted (red) position, dotted lines delineate the position of the axial canal (modified after Donovan, 1989); (F) stereom fields for Recent echinoderms (dark shaded area shows the stereom field indicative of muscles) (taken from Clausen & Smith, 2005); 1, fine labyrinthic stereom of articular surfaces of *Pleurocystites*; 2, coarse labyrinthic stereom of the interior of columnal of *Pleurocystites*; 3, fine labyrinthic stereom of articular surfaces of *Amecystis*; 4, coarse labyrinthic stereom of the interior of columnal of *Amecystis*.

The flexible stem is one of the most important features in glyptocystitids (Paul, 1984). It is divided into two parts. The expanded proximal portion with enlarged axial canal tapers away from the theca and bears alternating outer and inner columnals articulated synarthrially. The narrow distal portion, in turn, is characterized by homeomorphic, barrel-shaped columnals with a narrow lumen. While primitive glyptocystitids probably maintained the theca upright by means of the stem terminated by holdfast or tapered distally, more derived forms like pleurocystitids possessed a stem unattached distally and were thus probably motile (Paul, 1967; Fig. 1A). However, owing to the bizarre morphology of pleurocystitids, their mode of life has become a subject of controversy, mostly with respect to the posture, life position and mechanism of locomotion. For instance, based on burial taphonomy and theoretical consideration about functional morphology different authors suggested periproct-down (Paul, 1967; Sprinkle, 1974; Brower, 1999) or periproct-up (Bather, 1913) postures, and semi-infaunal (Brower, 1999), epifaunal with partly benthopelagic (hyperbenthic) lifestyles (Paul, 1967; Paul, 1984; Parsley, 1970; Sprinkle, 1974). Recent evidence for a *syn vivo* encrustation by edrioasteroids on the rhomb-bearing surface of pleurocystitid theca, strongly favours the periproct-down interpretation of the life posture and implies a fully epifaunal mode of life of pleurocystitids (Sumrall, 2000). However, the mechanisms responsible for the stem flexibility, still remain elusive. Two opposite models have been suggested. According to the first interpretation, the pleurocystitids contained muscles in the enlarged axial canal in the proximal part of the stem containing the fulcral ridge (Paul, 1967; Donovan, 1989). The second hypothesis implies that the stem flexibility was under the control of ligamentary mutable collagenous tissues (MCTs) and coeloms, which might have been expanded and contracted (Brower, 1999).

To test whether stem flexibility was under muscular or ligamentary control, we reconstruct the stem palaeoanatomy of two Ordovician pleurocystitid rhombiferans (*Pleurocystites* Billings, 1854 and *Amecystis* Ulrich & Kirk, 1921) using the observed link between skeletal microstructure and the nature of the infilling soft tissues in Recent echinoderms.

## Materials and Methods

Well-preserved specimens of two pleurocystitid rhombiferan species (*Pleurocystites squamosus* Billings, 1854 and *Amecystis laevis* Ulrich & Kirk, 1921) have been selected for microstructural investigation. The specimens are preserved as calcium carbonate and were collected from two lower Katian (Upper Ordovician) localities near Brechin, Ontario, Canada: (i) Carden Quarry (specimens derive from the boundary between Verulam and Bobcaygeon formations; GPS 44°34′16.93″N 79°6′6.44″W) and (ii) Tomlinson Quarry (specimens come from the Bobcaygeon formation; GPS 44°35′27.54″N 79°5′42.24″W).

The stem microstructure of well preserved proximal and distal columnals was investigated using a Scanning Electron Microscope (SEM) Philips XL-20 at the Institute of Paleobiology of the Polish Academy of Sciences in Warsaw. Additionally, 17 thin (mostly longitudinal) sections from different parts of the stem, polished down to 25 µm and coated with carbon, were investigated using optical and a cathodoluminescence (CL) microscope equipped with a hot cathode and Kappa video camera at the Institute of Paleobiology of the Polish Academy of Sciences in Warsaw. The following parameters were used for CL microscopy: an electron energy: 14 keV and a beam current: 0.1 mA. CL method was shown to enhance the stereom microstructure in recrystallized Paleozoic echinoderms (Gorzelak & Zamora, 2014; Gorzelak, Głuchowski & Salamon, 2014). Thickness of trabecular bars and dimensions of pores were measured directly from the SEM microphotographs.

The specimens are housed at the Institute of Paleobiology of the Polish Academy of Sciences in Warsaw (ZPALV.42O/1-30).

## Results

**Figure 2:**
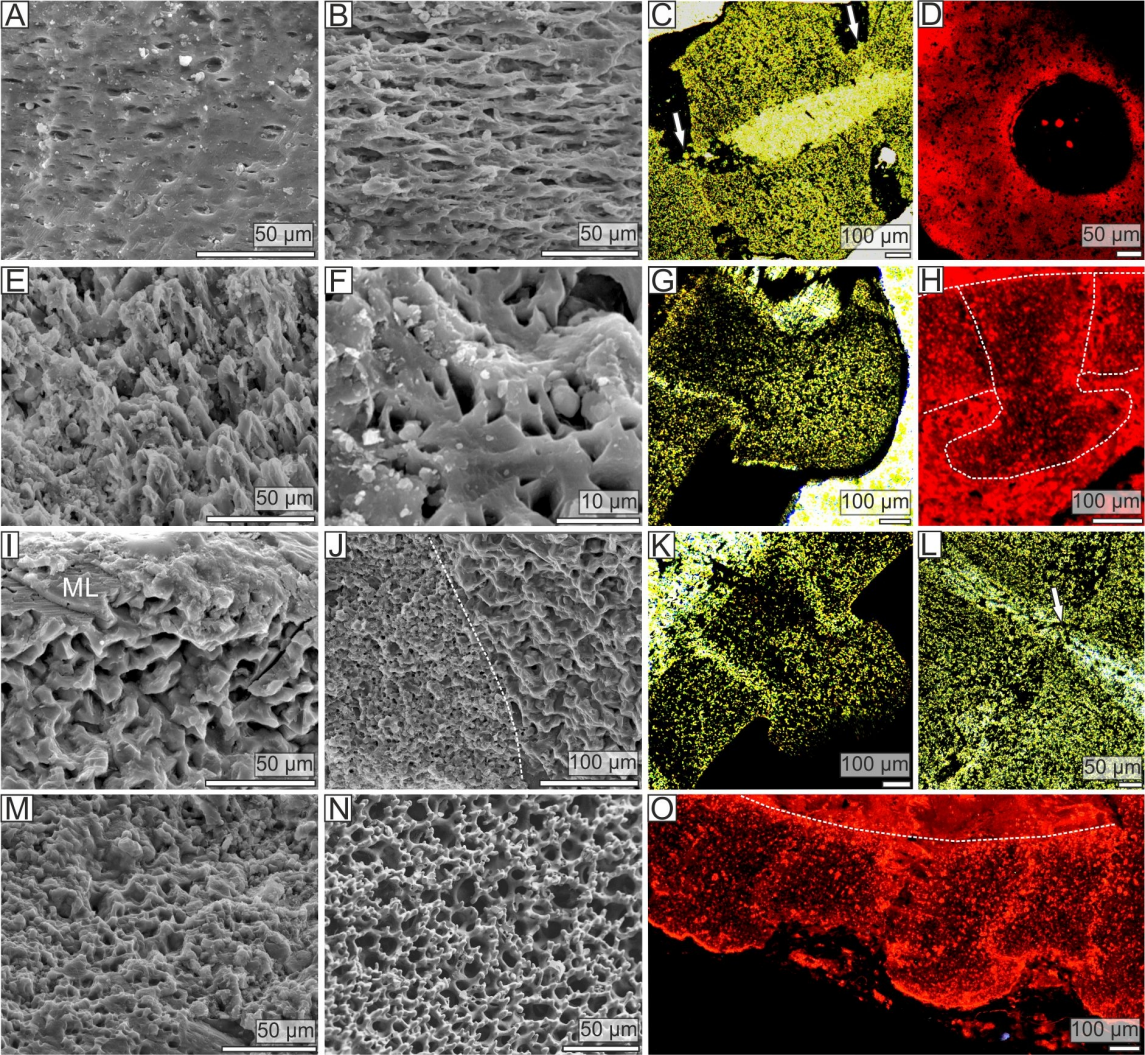
Stereom microstructure of the Late Ordovician pleurocystitids and a recent crinoid *Metacrinus rotundus*. Stereom microstructure of the Late Ordovician pleurocystitids from Canada (*Pleurocystites* (A–H) and *Amecystis* (I–M, O)) and a recent crinoid *Metacrinus rotundus* (N) from the Suruga Bay under SEM (A, B, E, F, I, J, M, N), optical (C, G, K, L) and CL (D, H, O) microscope; (A) microperforate layer of the distal columnal latera; (B) fascicular stereom near the lumen of the distal columnal; (C) longitudinally cross-sectioned distal columnals articulated with fulcrum (arrows) showing irregularly arranged trabeculae and pores displaying no alignment; (D) transversally cross-sectioned distal columnal showing irregularly arranged pores near the articular surface (E) fine labyrinthic stereom with needle-like projections on articular surface of distal columnal; (F) fine labyrinthic stereom on articular surface of distal columnal (G) longitudinally cross-sectioned proximal columnals showing irregularly arranged trabeculae and pores displaying no alignment; (H) longitudinally cross-sectioned proximal columnals (highlighted with dotted lines) showing irregularly arranged pores; (I) microperforate layer (ML) and coarse labyrinthic stereom of proximal columnal; (J) the contact (dotted line) between coarse and fine labyrinthic stereom near the articular surface of proximal columnal; (K) longitudinally cross-sectioned proximal columnals showing irregularly arranged trabeculae and pores displaying no alignment; (L) longitudinally cross-sectioned distal columnals articulated with fulcrum (arrow) showing irregularly arranged trabeculae and pores displaying no alignment; (M) fine labyrinthic stereom with needle-like projections on articular surface of proximal columnal; (N) fine labyrinthic stereom with needle-like projections of the muscle fields in brachial of Recent crinoid; (O) longitudinally cross-sectioned proximal columnals (the contact between cement is highlighted with dotted line) showing irregularly arranged pores.

The stem morphology and its microstructure in both pleurocystitid species is roughly similar. The inner proximal columnals are ring-like and possess a short fulcral ridge articulating to larger outer proximals having flanges externally (Figs. 1B and 1C). Distal columnals are cylindrical and display either synostosial (flat) or synarthrial (with fulcrum) articulation facets (Figs. 1D; 2C and 2L). The skeletal microstructure of investigated columnals is best revealed by SEM (Figs. 2A, 2B, 2E, 2F, 2I, 2J, 2M and 2N). The articular facet on each side of a fulcrum is mainly constructed of fine labyrinthic stereom, in which pores are irregular in size and display no alignment (Figs. 2E, 2F and 2M). In *Pleurocystites* trabeculae thickness and maximum pore diameter vary form 1 to 7 µm (mean = 3.7 µm; SD = 1.34; *N* = 20) and from 1 to 8 µm (mean = 3.8 µm; SD = 1.58; *N* = 20), respectively (Fig. 1F). In *Amecystis*, trabecular thickness (1–7 µm; mean = 3.7 µm; SD = 1.42; *N* = 20) and maximum pore diameter (2–9 µm; mean = 4.9 µm; SD = 1.77; *N* = 20) are roughly comparable (Fig. 1F). This labyrinthic stereom microfabric is sometimes associated with characteristic needle-like projections (Fig. 2E). The same structures, referred to as the “stalagmites,” have been identified in the muscle fossae of modern crinoids (Macurda & Meyer, 1975, plate 12: 5, 6). The interior of columnals in both species is mostly comprised of coarse labyrinthic stereom with irregular pores (*Pleurocystites*: 7–16 µm; mean = 10.6 ; SD = 2.39; *N* = 20; *Amecystis*: 7–17 µm; mean = 10.9; SD = 2.77; *N* = 20) and thick trabeculae (*Pleurocystites*: 7–15 µm; mean = 9.8; SD = 2.02; *N* = 20; *Amecystis*: 7–16 µm; mean = 9.2; SD = 2.21; *N* = 20) (Fig. 2I). The inner side surrounding the axial canal is usually constructed of a thin microperforated stereom layer or fascicular stereom (Fig. 2B). The latter stereom type is composed of near parallel trabecular rods (up to about 10 µm) that are branching and not forming continuous galleries. The columnal latera (Figs. 2A and 2I) is typically made of a microperforated stereom layer (up to about 25 µm thick). Under optical and CL microscope, the stereom types are somewhat less recognizable. However, irregularly arranged trabeculae and pores displaying no alignment are clearly visible (Figs. 2C, 2D, 2G, 2H, 2K, 2L and 2O).

## Discussion

Extensive studies on stereom microstructure in modern echinoderms revealed a strong link between the specific type of skeletal microstructure and the nature of infilling soft tissues. For instance, it has been shown that the ligaments commonly insert on galleried stereom, whereas the muscles are closely associated with the labyrinthic stereom (Smith, 1990). The only exception to this rule is a crinoid species *Calamocrinus diomedae* (Agassiz, 1890), in which short ligament fibrils may be also link to labyrinthic stereom (Holland, Grimmer & Wiegmann, 1991), although it appears to be more open and composed of coarse trabeculae (pore diameter and trabecular thickness > 10 µm). In all other crinoids, galleried and labyrinthic stereom are always associated with ligaments and muscles, respectively (Macurda & Meyer, 1975; Macurda, Meyer & Roux, 1978).

Pleurocystitid columnals reveal no galleried stereom perpendicular to the columnal facets that is indicative of the collagen ligament. Likewise, no straight stereom passageways passing through the columnals, that are diagnostic for the lateral through-going ligaments (Grimmer, Holland & Messing, 1984), are observed. Instead, fine and dense labyrinthic stereom with pore diameter and trabecular thickness falling to the field of the stereom associated with muscles in modern echinoderms was detected on the columnal facets (Fig. 1F). This strongly suggests that the pleurocystitid stem likely functioned as a muscular locomotory organ. The same stereom microfabrics, interpreted as muscle-bearing, have been documented in various fossil echinoderm ossicles (Lane & Macurda, 1975; Sevastopulo & Keegan, 1980; Clausen & Smith, 2005; Gorzelak, Głuchowski & Salamon, 2014). The presence of synarthrially articulating columns and a considerable flexure of pleurocystitid stem may also be suggestive of muscles. Indeed, the presence of transverse ridge in crinoid brachials is usually indicative of muscular articulations (Donovan, 1989). Likewise, it has been argued that post-mortem flexure is expected to occur in crinoid arms possessing muscles and that the crinoid arms bearing ligaments are typically preserved straight (Ausich & Baumiller, 1993).

Taken together, based on this line of evidence, re-evaluation of the previous hypotheses about the mechanism of locomotion in pleurocystitids can be made. The model implying that the flexibility of the stem in pleurocystitids was at least partly under the control of the ligamentary mutable collagenous tissues (MCTs) as in Recent crinoids can be rejected. In modern crinoids, articulations between columnals are always non-muscular (Donovan, 1989). The stem, however, may be flexible, especially when its articulation facets are synarthrial. The flexibility is enabled by MCTs, which may change their mechanical properties from stiff to soft under nervous control (Wilkie, 1996). However, the tissue conversion of MCTs proceeds slowly, within seconds to minutes, thus active swimming like a tadpole or even a slow crawling over the substrate in producing wagging or sinusoidal movements as hypothesized by various authors would not have been possible. Microstructural evidence strongly suggests that flexibility of pleurocystitid stem was enabled by muscles, not MCTs. This was earlier hypothesized by Paul (1967) and Donovan (1989) although according to their interpretations, the pleurocystitids contained muscles in the enlarged axial canal in the proximal part of the stem on the opposite side of the fulcral ridge. We demonstrate that the pleurocystitid stem indeed contained muscles, but they were also present in the distal portions of the stem and were rather solely associated with the articular facets. This is consistent with data on recent crinoids which show that the muscles are not present in the axial canal (Holland, Grimmer & Wiegmann, 1991). The enlarged axial canal in early pelmatozoans is thought to be largely filled by perihaemal fluids (Donovan, 2016). Thus, both hypotheses suggesting the presence of either muscles in the axial canal or MCTs on articular facet and possibility of coelom contraction are rather speculative.

Muscular synarthrial articulations of pleurocystitids certainly allowed rapid stem movements and enabled a free-living mode of life that was advantageous with respect to locomotion, predator avoidance and feeding. According to our model (Fig. 1E), bending of the stem might have been enabled by contracting and relaxing the muscles on the opposite side of the fulcrum. This enabled pleurocystitids to crawl over the substrate in producing sculling or sinusoidal movements of the stem from side to side. This hypothesis may be corroborated in the future with ichnological evidence which may provide useful insights into behaviour of enigmatic fossils (Rahman et al., 2009). Owing to the hydrodynamically efficient flattened body with the stem acting as a “tail” swimming is a possibility (Paul, 1967; Donovan, 1989); however, as hypothesized by some authors, was probably inefficient for pleurocystitids due to their heavily plated and dense skeletons (Parsley, 1970; Brower, 1999).

The proximal stem in glyptocystitids composed of inner and outer columnals already appeared in the Cambrian and is present in the most primitive representatives of the group such as *Ridersia* (Jell, Burrett & Banks, 1985) or *Sanducystis* (Zamora et al., 2016). This implies that those forms probably also had muscles in the stem although this needs to be tested in the future. However, early glyptocystitids are commonly preserved as molds (*Sanducystis* Zamora et al., 2016, *Barroubiocystis* Ubaghs, 1998) or are coarsely silicified (i.e., *Ridersia* Jell, Burrett & Banks, 1985). Thus, it is impossible to reconstruct the soft-tissue palaeoanatomy of these diagenetically altered fossils. The original function of the stem in early glyptocystitids was probably to maintain their unflattened thecae with few brachioles above the sea floor for a more efficient suspension feeding. Pleurocystitids, by contrast, likely used the stem to move in search of organic particles gathered from the substrate by means of only two flexible brachioles with food grooves open down toward the sediment (Brower, 1999). Glyptocystitids thus represent an echinoderm group that exemplified a switch from suspension to presumably detritus feeder (Parsley, 1970) and from sessile to vagile, explaining why these early echinoderm clades developed a flattened body plan.

## Conclusions

Evidence from the stereom microstructure strongly suggests that the stem of pleurocystitids fuctioned as a muscular locomotory organ. This is the first evidence for muscles in the stem of blastozoans. This discovery raises some questions concerning the origins of muscle-bearing stem in echinoderms as a whole. So far, the same labyrinthic stereom microfabrics indicative of muscles in echinoderm stems have been only documented in the Cambrian stylophorans (Stylophora) (Clausen & Smith, 2005), some undetermined Cambrian pelmatozoans (Clausen & Smith, 2008), and in a Devonian crinoid *Ammonicrinus* (Gorzelak, Głuchowski & Salamon, 2014). This strongly suggests multiple, independent origins of the muscle-bearing stems within Echinodermata.

## Additional Information and Declarations

### Data Availability

The following information was supplied regarding data availability: The research in this article did not generate any raw data.
